# Open urethroplasty versus endoscopic urethrotomy - clarifying the management of men with recurrent urethral stricture (the OPEN trial): study protocol for a randomised controlled trial

**DOI:** 10.1186/s13063-015-1120-4

**Published:** 2015-12-30

**Authors:** Rachel Stephenson, Sonya Carnell, Nicola Johnson, Robbie Brown, Jennifer Wilkinson, Anthony Mundy, Steven Payne, Nick Watkin, James N’Dow, Andrew Sinclair, Rowland Rees, Stewart Barclay, Jonathan A. Cook, Beatriz Goulao, Graeme MacLennan, Gladys McPherson, Matthew Jackson, Tim Rapley, Jing Shen, Luke Vale, John Norrie, Elaine McColl, Robert Pickard

**Affiliations:** Newcastle Clinical Trials Unit, Newcastle University, 1-2 Claremont Terrace, Newcastle upon Tyne, NE2 4AE, UK; University College London Hospital, 235 Euston Road, London, NW1 2BU, UK; Central Manchester Foundation Trust, Oxford Road, Manchester, M13 9WL, UK; St George’s Hospital, Blackshaw Road, London, SW17 0QT, UK; Academic Urology Unit, University of Aberdeen, Foresterhill, Aberdeen, AB25 2ZD, UK; Stepping Hill Hospital, Hazel Grove, Stockport, SK2 7JE, UK; Southampton General Hospital, Tremona Road, Southampton, SO16 6YD, UK; Lay member of the trial management group, ᅟ, ᅟ; Nuffield Department of Orthopaedics, Rheumatology and Musculoskeletal Sciences, University of Oxford, Windmill Road, Oxford, OX3 7HE, UK; Centre for Healthcare and Randomised Trials, Health Services Research Unit, University of Aberdeen, Foresterhill, Aberdeen, AB25 2ZD, UK; Institute of Cellular Medicine, Newcastle University, Framlington Place, Newcastle upon Tyne, NE2 4HH, UK; Institute of Health & Society, Newcastle University, Richardson Road, Newcastle upon Tyne, NE2 4AX, UK; The Medical School, Newcastle University, 3rd Floor William Leech Building, Framlington Place, Newcastle upon Tyne, NE2 4HH, UK

**Keywords:** Bulbar urethra, Urethral stricture, Randomised trial urethroplasty, Endoscopic urethrotomy, Cost-effectiveness analysis

## Abstract

**Background:**

Urethral stricture is a common cause of difficulty passing urine in men with prevalence of 0.5 %; about 62,000 men in the UK. The stricture is usually sited in the bulbar part of the urethra causing symptoms such as reduced urine flow. Initial treatment is typically by endoscopic urethrotomy but recurrence occurs in about 60 % of men within 2 years. The best treatment for men with recurrent bulbar stricture is uncertain. Repeat endoscopic urethrotomy opens the narrowing but it usually scars up again within 2 years requiring repeated procedures. The alternative of open urethroplasty involves surgically reconstructing the urethra, which may need an oral mucosal graft. It is a specialist procedure with a longer recovery period but may give lower risk of recurrence. In the absence of firm evidence as to which is best, individual men have to trade off the invasiveness and possible benefit of each option. Their preference will be influenced by individual social circumstances, availability of local expertise and clinician guidance. The open urethroplasty versus endoscopic urethrotomy (OPEN) trial aims to better guide the choice of treatment for men with recurrent urethral strictures by comparing benefit over 2 years in terms of symptom control and need for further treatment.

**Methods/Design:**

OPEN is a pragmatic, UK multicentre, randomised trial. Men with recurrent bulbar urethral strictures (at least one previous treatment) will be randomised to undergo endoscopic urethrotomy or open urethroplasty. Participants will be followed for 24 months after randomisation, measuring symptoms, flow rate, the need for re-intervention, health-related quality of life, and costs. The primary clinical outcome is the difference in symptom control over 24 months measured by the area under the curve (AUC) of a validated score. The trial has been powered at 90 % with a type I error rate of 5 % to detect a 0.1 difference in AUC measured on a 0–1 scale. The analysis will be based on all participants as randomised (intention-to-treat). The primary economic outcome is the incremental cost per quality-adjusted life year. A qualitative study will assess willingness to be randomised and hence ability to recruit to the trial.

**Discussion:**

The OPEN Trial seeks to clarify relative benefit of the current options for surgical treatment of recurrent bulbar urethral stricture which differ in their invasiveness and resources required. Our feasibility study identified that participation would be limited by patient preference and differing recruitment styles of general and specialist urologists. We formulated and implemented effective strategies to address these issues in particular by inviting participation as close as possible to diagnosis. In addition re-calculation of sample size as recruitment progressed allowed more efficient design given the limited target population and funding constraints. Recruitment is now to target.

**Trial registration:**

ISRCTN98009168 Date of registration: 29 November 2012.

**Electronic supplementary material:**

The online version of this article (doi:10.1186/s13063-015-1120-4) contains supplementary material, which is available to authorized users.

## Background

Men suffer urethral stricture because of scar formation in the urethral mucosa, which narrows the lumen. It is the commonest cause of difficulty in passing urine among younger and middle-aged men. The prevalence is approximately 200 per 100,000 men in their 20s rising to 900 per 100,000 men in their 70s, affecting about 62,000 men in the United Kingdom (UK) [1]. In the National Health Service (NHS) in England, urethral stricture in men results in 17,000 hospital admissions, 16,000 bed-days and 12,000 operations each year with an estimated cost of over £10 million [2]. Men seek help for urethral stricture because of progressive problems passing urine including reduced urine flow. The site and length of a stricture is characterised by endoscopic inspection and urethrographic imaging, and the degree of restriction to urine flow measured by maximum flow rate (Q_max_). The stricture is typically between one and five centimetres long and most (70 %) are located in the section of urethra that runs between the legs in the perineum (bulbar urethra). Symptomatic men with a bulbar stricture need surgical treatment to widen the narrowed section. The general standard approach in the UK for newly diagnosed strictures is endoscopic urethrotomy where a rigid endoscope with a steel blade attached to the end is passed into the urethra and the diseased segment is widened by incising it longitudinally through to healthy tissue. Cure rates for this first urethrotomy (defined as no recurrence within 2 years) are between 40 and 70 % [3].

The target population for this clinical trial is the 30–60 % of men with a bulbar urethral stricture who suffer recurrence after initial surgical treatment, since the best way to treat the recurrent stricture is uncertain. Repeat urethrotomy and open reconstructive urethroplasty are both reasonable options and the choice between the two is the focus of this study. Registry data suggests repeat urethrotomy is most often performed. It is minimally invasive, does not require specialist surgical expertise, and has a short period of catheterisation and recovery [2]. However, the rate of recurrence following the second (first repeat) urethrotomy is reported to range from 50 to 100 % (median 80 %) at 2 years [3]. Subsequent recurrences can lead to a chronic stricture state requiring repeated urethrotomies, on average every 2 years during a man’s lifetime [4]. Each urethrotomy carries a risk of adverse effects including protracted bleeding (6 %) and urinary tract infection (UTI; 10 %), potentially leading to loss of quality of life, unplanned hospital admissions and additional costs [5]. The alternative to endoscopic urethrotomy is reconstructive open urethroplasty. The urethra is approached through a skin incision in the perineum behind the scrotum. The narrowed segment is then identified and reconstructed typically using a patch of graft material to rebuild the diseased area and permanently widen the lumen [6]. The main perceived advantage of open urethroplasty is a potentially higher rate of long-term cure. A median success rate of 90 % freedom from stricture recurrence at 2 years has been reported in two systematic reviews of case series [6, 7] and is consistent between the UK and the United States (US) [8, 9]. Wound infection (5 %) and post-micturition dribble (10 %) are the most frequently occurring adverse effects together with pain at the graft donor site in the mouth [6, 10]. Registry data suggests that endoscopic urethrotomy remains the most frequently used treatment for men with recurrent urethral strictures. Annual hospital episode statistics from NHS England show that 818 urethroplasties of all types were carried out in 2013–2014 compared with 9,663 endoscopic urethrotomies/urethral dilations [2]. In the US, clinician surveys indicate that about 70 % of urologists would advise patients to proceed to repeat urethrotomy on initial stricture recurrence rather than consider urethroplasty [11]. Similarly, men undergoing urethroplasty in the UK have had a median of four previous urethrotomies [12]. In the UK and the US expert opinion has highlighted the perceived underutilisation of urethroplasty surgery [13]. The evidence base needed to decide whether urethroplasty is better than endoscopic urethrotomy for men with recurrent bulbar stricture is limited by lack of head-to-head comparison of the procedures in randomised controlled trials (RCTs) as documented by a Cochrane review [14].

At present, individual men have to make a trade-off between the invasiveness and presumed effectiveness of each operation. The OPEN trial seeks to answer the following question: in men with recurrent bulbar urethral stricture, does open urethroplasty result in better symptom control over 2 years than endoscopic urethrotomy and is its use cost-effective from the perspective of the UK NHS?

The null hypothesis is that the clinical effectiveness and cost-effectiveness of open urethroplasty is not different to endoscopic urethrotomy. The aim of this superiority design randomised trial is to have adequate power to assess whether open urethroplasty provides better quality of life at an affordable cost compared with the standard procedure of endoscopic urethrotomy.

## Methods/Design

This is a 50-centre, pragmatic patient-randomised two-arm superiority trial comparing, in parallel groups, open urethroplasty (experimental) against endoscopic urethrotomy (standard) for men with recurrent bulbar urethral stricture. The trial is set in a range of specialist and general UK NHS urology units. Patients and surgeons cannot be blinded to the allocated procedure. Central trial staff managing and preparing trial data for analysis will be blinded to allocated group.

### Inclusion and exclusion criteria

Patient eligibility criteria are as follows:

- Inclusion criteria
  * Adult males aged 16 years or greater.
  * Stricture located predominantly in the bulbar urethra.
  * Undergone at least one previous intervention (endoscopic urethrotomy, urethral dilatation, urethroplasty) for bulbar urethral stricture.
  * Clinician and patient agreement that intervention is required.
  * Suitable for general or regional anaesthesia of up to 3 hours’ duration.
  * Willingness to have up to a 2-week period of catheterisation.
  * Provide written informed consent for participation in the study prior to any study-specific procedures.

- Exclusion criteria
  * Perineal sepsis and/or fistula.
  * Not suitable for up to a 3-hour period of anaesthesia, or inability to adhere to the trial protocol due to co-morbidity.
  * Inability to provide informed consent to randomisation.
  * Previous participation in this study.

### Trial interventions

Two surgical interventions will be investigated:

1. Urethrotomy (standard)
  The standard intervention of endoscopic urethrotomy typically takes 45 minutes under general anaesthesia, with prophylactic antibiotic cover. With the patient’s legs partially elevated in supporting stirrups, the endoscope is passed along the lubricated penile urethra to locate the distal end of the stricture. A fine-calibre wire guide is then passed through the stricture to the bladder. Using this guide, the stricture is progressively divided longitudinally using the mounted scalpel in the dorsal ‘12 o’clock’ orientation until the proximal end of the stricture is reached. For short flimsy strictures, dilatation with the instrument may suffice without making a cut. The instrument is withdrawn and a 16 French calibre silicon catheter inserted through the urethra to the bladder and left on free drainage [15]. The patient recovers on the ward and is discharged according to local practice (NHS median stay is 1 day) usually with the catheter still in place. He returns to hospital after an interval or remains as an inpatient according to local practice (typically 2–3 days) for catheter removal and voiding check and then is followed up by outpatient review and urinary flow rate at 3, 12 and 24 months. According to the clinician’s policy at individual recruiting sites, and patient choice, participants may continue a programme of intermittent self-dilation (ISD) using a plastic catheter following urethrotomy.

2. Urethroplasty (experimental)
  Open urethroplasty involves the reconstruction of the urethra through an appropriately sited longitudinal skin incision made in the perineum beneath the scrotum between the legs. It requires pre-operative X-ray urethrography. The surgery takes 2–3 hours under general anaesthesia with the patient’s legs partially elevated in supports and prophylactic antibiotic given. The bulbar urethra is located through the skin incision and mobilised. The strictured segment is incised longitudinally with the cut extending into visibly healthy urethra proximally and distally. For the majority of cases where the stricture is relatively long and dense, a graft of oral mucosa is inserted to widen the strictured area of urethra (patch urethroplasty) [16]. The graft (typically 5 cm by 2 cm) is harvested according to a standard technique from the inner cheek with the donor site left open to heal spontaneously or closed with sutures according to surgeon practice [17]. The graft is prepared, positioned appropriately, sutured to the cut urethral edges, and stabilised on the deeper tissues within the perineal wound. This incorporates the graft mucosal surface into the lumen of the urethra. For short supple strictures, simple excision of the scarred area and re-joining of the cut ends may be performed without a graft (anastomotic urethroplasty) [18]. A 16 French calibre silicon catheter is then passed to the bladder and left in situ on free drainage. The patient recovers on the ward before discharge home (NHS median stay is 2 days). The patient returns after an interval according to local practice (typically 2–3 weeks) for an X-ray urethrogram to check leak-free healing, and catheter removal. Follow-up comprises outpatient review and urinary flow rate at 3, 12 and 24 months. Some surgeons prefer a further urethrogram or endoscopic examination to be performed at about 12 months to ensure that no recurrence has occurred.
  Surgeons performing the procedures will be UK consultant, accredited urologists or senior urology trainees under consultant supervision. Endoscopic urethrotomy is a procedure that all operating urologists have competency for and will take place at the hospital site where the participant was randomised. Open urethroplasty is a specialised procedure carried out for trial participants by urologists recognised as being urethral surgeons by their employing NHS organisation. The surgery will be carried out at regional referral centres (which are all registered sites for the OPEN trial) to which participants will be referred using established routine NHS pathways for undertaking this surgery.

### Identification and screening of participants

Participants will be identified by NHS clinical staff (principally consultant and trainee urologists) at participating centres. They will either be referrals from primary care or men already under review in urology clinics. The clinician will outline the OPEN trial and ask the patient if they are willing to discuss participation. If they want to know more, they will be referred to the local research team for eligibility assessment on the same day or at a mutually convenient time within 2 weeks.

A member of the local research team will complete a trial screening form using information from the prospective participant and from the clinical record to document fulfilment of the entry criteria. If the patient is eligible and in provisional agreement to participate, a local research team member will meet with the patient either immediately in the clinic or within 4 weeks at a mutually convenient time and place to discuss the trial further. This initial meeting can take place by telephone.

### Recruitment procedures

Eligible participants who express interest in participating in the OPEN trial will have the study explained to them by local research staff and will be given a patient information pack to read. They will be informed that the local research team will contact them using their preferred means of contact within 7 days to find out whether or not they would like to take part, or alternatively to provide further information. If they agree to take part, they will be invited to meet with local research staff to give written consent to be randomised between open urethroplasty and endoscopic urethrotomy. The timing of randomisation will be according to patient and clinician wishes and local arrangements in terms of waiting times and operating theatre list planning. These factors will be balanced as far as possible to ensure that surgery takes place as soon after randomisation as is acceptable to participants and the treating NHS organisation. The trial will use existing routine local arrangements concerning pre-assessment, admission, consent for surgery, conduct of surgery and after care. We will also ask men to take part in a qualitative sub-study using semi-structured audio-recorded interviews. This will be first to assess the effect of treatment preferences on recruitment during the initial phase of the study and second to perform a time trade-off (TTO) experiment during the main trial phase.

A screening log will be kept by local research staff to document details of subjects invited to participate in the study. Non-identifying patient details to allow assessment of selection bias such as age, number and type of previous procedures, recruitment site and length of stricture will be uploaded to a secure study website for analysis. For subjects who decline participation, this will document reasons for non-participation.

### Consent procedures

Informed consent for randomisation will be undertaken by appropriate staff at the trial sites as detailed in the site delegation log. This will include medical staff and research nurses involved in the study who will give time for participants to ask any questions they may have following review of the trial information pack. Following receipt of information about the study, participants will be given at least 48 hours and up to as much time as they need to decide whether or not they would like to participate. Those wishing to take part will provide written informed consent by signing and dating the study consent form, which will be witnessed and dated by a member of the research team with documented, delegated responsibility to do so. Written informed consent will always be obtained prior to randomisation and with additional clinical consent prior to study-specific interventions. The participant will specifically consent to their general practitioner (GP) being informed of their participation in the study. The right to refuse to participate without giving reasons will be respected.

### Randomisation process

Eligible and consenting participants will be randomised to one of the two intervention groups using the 24-hour telephone Interactive Voice Response randomisation application or via the web-based application, both hosted by the Centre for Healthcare Randomised Trials (CHaRT) at The University of Aberdeen, UK. The randomisation algorithm will use recruitment site and time since last procedure (<12 months or ≥12 months) as minimisation factors to allocate treatment to intervention and standard groups in a 1:1 ratio. A random element (20 %) will be incorporated into the randomisation algorithm, so that one in five allocations will be randomly switched. The Principal Investigator (PI) at site, or individual with delegated authority, will access the telephone or web-based system when an eligible participant has consented to participate in the study. Patient screening identification, initials, recruiting site and time since last intervention (stratifying variables) will be entered into the voice-activated or web-based system, which will return the allocation status. Participants will be informed of their allocated treatment group following randomisation and arrangements will be made to carry out the procedure according to the standard process of care at the recruiting site.

### Subject change of status (including withdrawal of consent)

Patients will remain on the study unless they prospectively withdraw consent for any further involvement in the study or in the unlikely event that the PI, Chief Investigator (CI) or trial office feel it is no longer appropriate for the patient to continue. Participants cannot retrospectively withdraw consent, and any data collected up to the point of withdrawal of consent can be used in the analysis. If a participant wishes to change their status in respect of full participation in the trial we will ask to continue collecting outcome data both by posting them symptom and quality of life questionnaires for the primary outcome, and from their clinical records. We will, within the consent and ethics framework, seek to complete trial follow-up documentation as fully as possible in line with our pre-stated intention-to-treat primary analysis. Should a patient decide to completely withdraw from the study, all efforts will be made to report the reason for withdrawal as informatively as possible.

### Trial procedures

Participants will complete patient-reported outcome measure (PROM) questionnaires at baseline and throughout their trial participation. The questionnaire consists of a six-item urinary symptom score, pictorial urine flow question, single-item condition-specific quality of life questions and the EuroQoL five-dimension questionnaire (EQ-5D). The baseline and pre-intervention PROMs will be completed in hospital. A PROM will also be provided to the participants at discharge from the hospital to complete 1 week after catheter removal. Further questionnaires will be mailed to participants at 3, 6, 9, and 12 months post intervention, at 18 and 24 months post randomisation, and at the end of the study (December 2017). The timing of follow-up points initially from intervention, and then later in the observation period from randomisation, ensures that participants complete an adequate number of questionnaires whilst allowing for the time between randomisation and undergoing the intervention. Participant costs questionnaires (PCQs) will be posted to patients at 6, and 12 months post intervention, and at 18 and 24 months post randomisation. Up to two reminders may be sent where participants fail to return the questionnaires. In addition, participants will be reviewed in clinic at 3 months post intervention and at 24 months post randomisation for adverse event review and a flow rate measurement. A further adverse event review will also take place at 12 months post intervention, either in clinic or during a telephone review. Case report forms (CRFs) for each visit will be completed by the research team. If a participant has a re-intervention whilst in the 24-month post randomisation follow-up period this data will be collected on an additional CRF, and patients will be asked to complete both a pre-intervention and 1-week post catheter removal PROM.

### Study objectives

- Primary objectives are:
  * To determine the relative impact on trajectory of symptom control as measured by the International Consultation on Incontinence Modular Questionnaire Male Short Form (ICIQ-Male SF) voiding symptom questionnaire over 24 months after randomisation.
  * To determine the incremental cost per quality-adjusted life year (QALY) at 24 months after randomisation.

Secondary objectives are:

- Clinical:
  * To determine the relative change in urinary flow rate at 24 months.
  * To determine the relative rate of recurrence and need for re-intervention up to 24 months.
  * To establish the safety profile of each procedure.
  * To determine the relative impact of symptom control and quality of life over the total study duration (summarised as median time since intervention).
  * To determine the relative rate of need for re-intervention over the total study duration (summarised as median time since intervention).

- Economic:
  * To estimate the incremental cost per QALY modelled over 10 years.

### Primary effectiveness outcome measure

Difference in trajectory of symptom control measured by area under the curve (AUC) of the ICIQ-Male SF symptom score [19] completed by participants during routine clinic visits or by postal or online questionnaires at baseline, immediately prior to surgery, 1 week after catheter removal, at 3, 6, 9, 12 months after intervention, and at 18 and 24 months after randomisation, and additionally prior and subsequent to any surgical re-intervention. The ICIQ-Male SF modular questionnaire has been validated in this patient group [20]. The trial duration allows for follow-up of all men at 24 months after randomisation. We will also collect longer-term follow-up data for those men who undergo their allocated intervention earlier during the recruitment window by completion of additional outcome questionnaires at 24 months post surgery and at the end of the study (December 2017).

### Secondary effectiveness outcome measures

1. Difference in condition-specific quality-of-life (QoL) trajectory measured by the AUC for the single item ICIQ-Male SF QoL score recorded by participant questionnaire completed during clinical visits or by post or online at baseline, immediately prior to surgery, 1 week after catheter removal, at 3, 6, 9, 12 months after intervention, and at 18 and 24 months after randomisation, and additionally prior and subsequent to any surgical re-intervention.
2. Difference in global sexual functioning trajectory measured by the AUC for the single-item International Index of Erectile Function (IIEF) male sexual QoL score recorded by participant questionnaire completed during clinical visits or by post or online at baseline, immediately prior to surgery, 1 week after catheter removal, at 3, 6, 9, and 12 months after surgery, and at 18 and 24 months after randomisation and prior and subsequent to any surgical re-intervention.
3. Difference in generic QoL trajectory measured by the AUC for the EQ-5D five-level version (5 L) version total score based upon responses to five-dimension items and using UK population valuations (0 death to 1 full health) and visual analogue scale score (0 worse possible health state to 100 best possible health state). The EQ-5D will be included in participant questionnaires administered during routine clinical visit or by postal or web-based participant questionnaire at baseline, immediately prior to surgery, 1 week after catheter removal, at 3, 6, 9 and 12 months after intervention, 18 and 24 months after randomisation, and prior and subsequent to any surgical re-intervention.
4. Difference in rate of improvement of urinary flow rate measured as part of routine care at baseline, 3 and 24 months with an increase in Q_max_ ≥10 ml/s from baseline taken to signify a successful outcome [21].
5. Difference in rate of recurrence and need for further intervention recorded from the clinical record for those returning to the care of their original specialist with recurrent stricture, by patient questionnaire for participants seeking care elsewhere, and checked by the local trial research staff at 24 months after randomisation. Questionnaires regarding further intervention will be sent to participants at 24 months after their initial surgery (if this falls before the end of the study) and also at the end of the study (December 2017). For participants in whom the clinical record documents stricture recurrence but no further intervention has occurred, the relevant clinical information will be sent in anonymised form as a case vignette to an expert panel of five urology clinicians independent of the trial to determine whether or not there is a majority opinion that clinical recurrence of the stricture has been confirmed.

### Primary cost-effectiveness outcome measure

The primary cost-effectiveness outcome is cost-utility measured as incremental cost per QALY at 24 months. Utility measured by QALYs will be obtained from EQ-5D 5 L administered at the time intervals described above. Total QALY gain for each group will be calculated by the AUC method. QALYs from short-term impacts immediately following interventions will be estimated from a sub-study using the TTO technique administered in a structured interview. This will provide a more accurate estimate of QALYs than that based solely on the administration of the EQ-5D over the follow-up period. Associated costs will be those resources required to deliver trial interventions, and costs incurred during follow-up for condition-related primary and secondary care use. Intervention-related costs will be based on participant-level data (e.g. operating time, grade of surgeon and protocol deviations), other related hospitalisations and secondary interventions provided in hospital, outpatient visits (number and speciality) obtained from clinical records and the CRF. Unit costs for intervention-related resource use will be calculated according to NHS reference cost schedules [22] or based on study-specific estimates from participating centres. Costs incurred during follow-up will be estimated based on information collected in the PCQ. Use of primary care service (number and type of GP visits), secondary care service (hospital outpatient visits and inpatient stay following surgery) and other healthcare service (e.g. patients’ out-of-pocket health expenses, use of private healthcare) will be collected from part A of the PCQ completed at 6 and 12 months after initial surgery, and at 18 and 24 months after randomisation. Patient time and travel costs and other related societal costs (e.g. time off work) will be obtained from part B of the PCQ completed at 6 months after initial surgery. Unit costs of healthcare services and medications will be based on published sources, such as the NHS reference Healthcare Resource Group (HRG) tariffs, the British National Formulary [23] and Personal Social Services Research Unit [24].

### Secondary cost-effectiveness outcome measures

1. Difference in mean costs calculated from the NHS perspective and from the societal perspective [both NHS and individual patients)].
2. Longer-term cost-utility reported as incremental cost per QALY at 10 years. Costs and QALY up to 24 months will be based on trial data and those beyond 24 months will be modelled from the literature for each trial intervention

### Safety

- For purposes of this protocol:
  * All adverse events will be recorded as they occur at and around the time of primary or re-intervention surgery, and by recall at 3 months, 12 months post surgery and 24 months post randomisation.
  * Any serious adverse events will be recorded throughout the duration of the trial.
  * Serious adverse events exclude any pre-planned hospitalisations (e.g. elective surgery) not associated with clinical deterioration.
  * Serious adverse events exclude routine treatment or monitoring of the studied indication, not associated with any deterioration in condition.
  * Serious adverse events exclude elective or scheduled treatment for pre-existing conditions that did not worsen during the study.
  * Serious adverse events exclude stricture (symptom or urine flow) recurrence which is already documented and monitored within study.

### Recording and reporting serious adverse events or reactions

#### Summary

Research team staff at individual sites will complete the adverse events (AE) section of the relevant CRFs and input the details of the AEs into the database hosted by the Centre for Healthcare and Randomised Trials (CHaRT) in Aberdeen, UK via the secure study website. The CHaRT web-based system will automatically alert the Trial Manager based in the Clinical Trials Unit, Newcastle University, Newcastle upon Tyne, UK if any new AEs are added or any amendments are made to the data in existing ones. The trial management office in Newcastle in conjunction with the Chief Investigator (CI) will have the responsibility for any decision making and forward reporting of AEs. All AEs should be recorded in the CRF. Suspected unexpected serious adverse events (SUSARs) that are considered to be causally related will be separately reported on the specific form. Adverse events that are serious but expected will not be reported on the SUSAR form.

##### Adverse events (AEs)

All AEs during study participation will be reported on the study CRF and entered by local investigators into the trial web management system. The individual investigator at each site will be responsible for managing all AEs according to local arrangements.

##### Serious adverse events (SAEs)

All unexpected SAEs that are related or of uncertain causality during study participation shall be reported to the CI through the study website within 24 hours of the site learning of its occurrence. The initial report will be made by completing the electronic SUSAR form which will automatically send e-mail notification to the Trial Manager and CI in Newcastle. In the case of incomplete information at the time of initial reporting, all appropriate information should be provided as follow-up as soon as this becomes available. Relationship of the SAE to trial participation should be assessed by the investigator at site, as should the expected or unexpected nature of the AE. The Research Ethics Committee (REC) will be notified by the CI (on behalf of the Sponsor; The Newcastle upon Tyne Hospitals NHS Foundation Trust) of all SUSARs within 15 days of the CI becoming aware of the SUSAR (unless urgent safety measures are required, in which case initial notification by telephone will be made immediately the CI becomes aware of the AE, with notice in writing following within 3 days). SUSARs will be reported using the National Research Ethics Committee (NRES) Report of Serious Events Form, version 3, April 2007 [25]. The CI will ensure that the Sponsor is notified of any SUSARs in accordance with local trust policy. Local investigators should report any SUSARs as required by their local NHS Trust Research and Development Office.

### Statistical analysis

The primary outcome, AUC for the ICIQ-Male SF symptom score, will be generated for each participant using the trapezoidal rule. Symptom score data for participants who have missed a scheduled time point will be estimated using a multiple imputation approach to make use of partial outcome data [26]. Sensitivity analyses will be conducted to assess the robustness of the treatment effect estimate to these approaches. The primary outcome measure will be analysed using linear regression with adjustment for the minimisation variables [site of recruitment and time since last procedure (<12 months or ≥12 months)]. The main statistical analyses will be based on all participants as randomised, irrespective of subsequent compliance with the treatment allocation. Secondary outcomes will be analysed using generalised linear models with adjustment for minimisation and baseline variables as appropriate. All estimates will be presented with 95 % confidence intervals. Subgroup analyses will explore the possible modification of treatment effect by clinically important factors; time since last procedure (<12 months or ≥12 months) as a global measure of stricture severity, age, stricture location, and length. This will be done by including treatment-by-factor interactions in the model. All analyses will initially be performed on an intention-to-treat basis. We will consider additional analyses such as per-protocol depending on the levels of compliance with allocated treatment. Further analysis will explore the impact of variations in treatment delivered; such as use of anastomotic urethroplasty, use of intermittent self-dilatation after urethrotomy, and delay between decision to treat and undergoing the intervention. From the initial phase of recruitment we will report estimates of recruitment rates and potential participant availability, together with appropriate confidence intervals. There are no planned interim outcome analyses; all analyses will occur following completion of trial follow-up. Interim analyses will be performed if requested by the Data Monitoring and Ethics Committee (DMC). All analyses will be outlined in detail in a pre-specified statistical analysis plan.

### Sample size calculation

We initially planned to recruit 500 participants to the study. This used the assumption that the standard deviation (SD) of the primary outcome measure (ICIQ-Male SF questionnaire score 0–24) would be 0.3. Using this figure with 90 % power (two-sided 5 % significance level), 235 participants per group (470 in total) would have been required. This would equate to being able to detect at least a 0.1 difference in the AUC on the standardized 0–1 utility scale, assuming a SD of 0.33 or less. The SD of the ICIQ-Male SF symptom AUC in a previous study was 0.15 [20]. Given our lack of more precise information, we initially conservatively allowed for a larger SD in recognition of the less selected, more pragmatic (i.e. more representative) population to be recruited to this trial and the shorter follow-up period in the previous study. Such a difference in symptom burden and associated QoL has been observed in different clinical areas for health-related quality-of-life (HRQoL) measures [27]. In terms of treatment effect size, this is in the small to medium range as observed in other clinical studies [28]. To allow for the anticipated approximately 5 % of participants for whom outcome data is completely missing, and therefore the AUC cannot be calculated, it was initially proposed to randomise 500 participants. The slower than initially expected recruitment rate has allowed us to use blinded trial outcome data to provide a more precise estimate of the SD. We have calculated the SD of the patient-reported symptom score (primary outcome measure) from 69 active OPEN participants who have submitted at least one post-operative measure (220 measurements in total). The re-calculation gives an SD value of 0.165, which reduces to 0.15 when adjusted for baseline score and centre, considerably smaller than the assumed value of 0.33, which was used in our initial calculations. Using this SD in our sample size calculation would indicate a target population of 96 participants. However, no 18 or 24 month data are currently available and variability would be expected to increase over time. Allowing for this and other factors increasing variability we have assumed a SD of 0.21 or less. This would require 186 randomised men with complete follow-up inflated to 200 in total to allow for loss to follow-up. Based on findings from recruitment to the ProtecT trial, which randomises between surgery and less invasive options for men with localised prostate cancer, we originally conservatively estimated a 55 % agreement to participate rate amongst those eligible requiring 910 men to be approached [29]. We have reassessed this in the light of slower than anticipated recruitment rate from screening data from the trial now available to us showing a 22 % agreement to participate rate. For the qualitative interview study we estimate, based on previous experience in such methodology, that up to 10 men who agree, and up to 10 men who do not agree to be randomised and up to 12 purposively sampled specialist and general urologists will be required to achieve data saturation for thematic analysis. The trial is also secondarily powered to determine whether the use of urethroplasty will result in a 30 % (from 50 % to 20 %) reduction in need for further intervention at 2 years compared to urethrotomy as a secondary outcome. To detect this difference using the binomial test of proportions with 90 % power at the 5 % significance level would require 48 men to complete the study in each arm.

### Economic analysis

For the within-trial analysis the primary measure of effectiveness will be incremental QALY gain based on responses to the EQ-5D over a 24-month time horizon. Cumulative mean costs to patients and the NHS over the trial follow-up period for each intervention will be estimated from patient and NHS resource use data collected during the trial and their unit costs based on standard sources. The difference between mean costs between the two arms will be combined with their relative effectiveness to produce an incremental cost per QALY gained at 24 months. Methods such as bootstrapping will be used to produce confidence intervals around difference in costs and effects. The same method will be used to produce cost-effectiveness acceptability curves. Sensitivity analysis will be used to explore other uncertainties such as alternative cost estimates. Open urethroplasty is expected to be both more effective and more costly than endoscopic urethrotomy, and its benefits may persist beyond 24 months. Therefore, we will conduct a cost-utility analysis using a Markov model with 10-year time horizon to compare incremental cost, incremental QALY and incremental cost per QALY of the interventions. The model structure will be based upon care pathways mapped out by the project team and the literature. Guidelines for best practice for modelling will be followed [30]. Trial data will be a vital source to populate the model, and additional data on care and events beyond 24 months will be based on structured literature review. Specific trial data will include effectiveness and costs of the interventions and ongoing care for participants whose strictures recur. Utility scores will be calculated based on responses to the EQ-5D from participants whose strictures do not require re-intervention, and from those having AEs. These will be cross validated with existing values [31]. We will also conduct extensive probabilistic sensitivity analyses by attaching appropriate distributions to the model input parameters. The results of these analyses will be presented as cost-effectiveness acceptability curves. Deterministic sensitivity analyses will be used to explore other forms of uncertainty such as varying the model’s time horizon. As the duration of follow-up in both the within-trial and the model-based analyses is greater than 1 year both costs and effects will be discounted at 3.5 % [32].

### Ethics and regulatory issues

The conduct of this study will be in accordance with the recommendations for physicians involved in research on human subjects adopted by the 18th World Medical Assembly, Helsinki 1964 and later revisions. Favourable ethical opinion to carry out the research across all UK NHS organisations was granted by the UK National Research Ethics Committee North East - Newcastle and North Tyneside 1 (reference 12/NE/0343). The Research and Development (R&D) Department of The Newcastle upon Tyne Hospitals NHS Foundation Trust is the Trial Sponsor. Local approvals will be sought before recruitment may commence at each site. The Study Coordination Centre will require a written copy of local approval documentation before initiating each centre and accepting participants into the study. Information sheets will be provided to all eligible subjects and written informed consent obtained prior to any study procedures.

## Discussion

There is continued uncertainty amongst clinicians and men suffering recurrent bulbar urethral stricture whether urethroplasty or urethrotomy represent the best treatment with no new trials identified in a recently updated Cochrane review [14]. This in turn makes it difficult for providers of healthcare to plan appropriate services. The OPEN trial therefore remains necessary to provide the required evidence of effectiveness and cost-effectiveness.

The timely recruitment of sufficient numbers of trial participants has been the key challenge to date (June 2015) for the OPEN trial. The incidence of recurrent bulbar urethral stricture is relatively low, so identifying potential participants has been difficult. Furthermore, eligible patients have the option of either treatment in standard care (urethrotomy or urethroplasty), and as a result they are more inclined to follow their individually preferred treatment pathway rather than accepting randomisation. This may be exacerbated by clinicians stating that one option is better than another. In the screening data collected to date, 66 % of potentially eligible patients declined entry to the trial due to having a preference for either treatment.

Data collection through return of PROMs has remained high throughout the trial (75–100 % return rate for differing time points). This is attributed to strategies designed to encourage men to return their PROMs; e-mail and/or text pre-notification of PROM delivery, mailed and telephone reminders of overdue PROMs, and monetary ‘thank you’ gifts (£25 gift voucher after randomisation, £25 gift voucher at 24 months after randomisation, and a further £25 gift voucher after completion of all follow-up data). The lowest return rate for the PROMs was at the 1-week post catheter removal time point (currently at 68 % return). These were initially sent by post to the patient after their surgery, which relied on the sites uploading the dates of surgery and catheter removal into the e-CRF immediately. Sites were then encouraged to give the patient these PROMs at discharge, which has caused an improvement in the return rates.

Waiting times for surgery vary between each participating centre, and between each arm of the trial. For this reason, two anchor points were decided upon for follow-up analysis; for the primary analysis anchoring follow-up to a fixed 2-year post randomisation point will allow for analysis of symptom control and quality of life from the initial decision to treat, providing a pragmatic approach to account for the difference in waiting times for each intervention. As a secondary outcome we will additionally analyse symptom control and quality of life over the total study duration (summarised by median time since intervention) giving additional information on longer duration of follow-up for men after undergoing the studied interventions.

### Strategies used to optimise identification and randomisation of men willing to participate

High-quality randomised clinical trials remain essential to clarify the comparative effectiveness of treatments for conditions where differing therapeutic options are possible. However recruitment is often challenging, particularly when the options being compared are both available as is the case for the OPEN trial. As the trial has progressed we have used a number of strategies to ensure that men eligible for inclusion and who are willing to take part have the opportunity to participate in OPEN.

1. *Inclusion and interpretation of feasibility phase*: the pre-planned feasibility phase of OPEN allowed realistic estimate of the required duration of recruitment period and the optimum number of centres required to be set up. Detailed discussion of the issues and potential solution with the funder facilitated necessary changes to trial design allowing continuation.
2. *Completion of qualitative work to establish factors determining willingness of patients and support of their clinicians to consider participation*: timely and successful completion of a planned qualitative study first established that the aims of the trial were important to men eligible to participate given the troublesome and chronic nature of their symptoms and reinforced the rationale and need for the trial [33]. As part of this work, we found that men eligible for inclusion were most likely to be willing to participate when their symptoms had first recurred and this was the point at which they expressed most uncertainty as to which option would be best for them as individuals. Both general and specialist clinicians were also very supportive of the aims of the trial given the uncertainty of guidance on best treatment, but expressed concerns regarding delivery of balanced information to men eligible for participation. Appropriate written guidance and an example video were provided to assist supported by personal contact from the trial team.
3. *Establishment of general urology centres as recruiting sites*: most men eligible for the trial present initially to general urology units prior to potential referral to specialist urethral surgeons. This was established as the main focus of recruitment as the results of the trial will in future inform decision making around referral. We selected sites with a motivated urologist Principal Investigator and encouraged realistic prediction of likely number of men identified and consenting to randomisation. Visits by the Chief Investigator to go through local difficulties were offered and carried out.
4. *Sharing of experience*: representatives from all sites were invited to take part in regular telephone conferences facilitated by the central trial team and bi-monthly e-newsletters were circulated to encourage sharing of local difficulties and their solutions.

### Trial status

The first participant was recruited in February 2013. The trial is currently open to recruitment in 50 UK centres. Recruitment is scheduled to finish in December 2015 with follow-up complete at December 2017. Up to the end of November 2015; 214 participants have been enrolled against the target of 210. In line with current trial protocol reporting recommendations we include a SPIRIT checklist (Additional file 1) and administrative details concerning trial conduct (Additional file 2).

## Additional files

Additional file 1:
**SPIRIT 2013 checklist: recommended items to address in a clinical trial protocol and related documents*.** (DOC 120 kb) Additional file 2:
**Administrative details.** (DOCX 27 kb)

## Abbreviations

AE: Adverse event
AUC: Area under the curve
CHaRT: Centre for Healthcare Randomised Trials, University of Aberdeen
CI: Chief Investigator
CRF: Case report form
DMC: Data Monitoring and Ethics Committee
EQ-5D-5 L: EuroQoL five-dimension questionnaire (five-level version)
GP: General practitioner
HRQoL: Health-related quality of life
ICIQ-Male SF: International Consultation on Incontinence Modular Questionnaire Male Short Form
IIEF: International Index of Erectile Function
NHS: National Health Service
PCQ: Participant costs questionnaire
PI: Principal Investigator
PROM: Patient-Reported Outcome Measure
Q_max_: maximum flow rate
RCT: Randomised controlled trial
REC: Research Ethics Committee
QALY: Quality-adjusted life year
QoL: Quality of life
R&D: Research and Development Departments of NHS Trusts
SD: Standard deviation
SAE: Serious adverse event
SUSAR: Suspected unexpected serious adverse reaction
TTO: Time trade-off
UTI: Urinary tract infection
