# Supplementary material for: Open urethroplasty versus endoscopic urethrotomy - clarifying the management of men with recurrent urethral stricture (the OPEN trial): study protocol for a randomised controlled trial
Source: Trials. 2015 Dec 30;16:600. doi: 10.1186/s13063-015-1120-4 (PMC4697334; doi:10.1186/s13063-015-1120-4)
Supplement: Additional file 2: — Administrative details. (DOCX 27 kb) [file 13063_2015_1120_MOESM2_ESM.docx]

**Administrative Details**

Title

**Op**en urethroplasty versus **en**doscopic urethrotomy - clarifying the management of men with recurrent urethral stricture (The OPEN Trial): study protocol for a randomized controlled trial

Trial Registration

International Standard Randomised Controlled Trial, number 98009168

UK National Institute for Health Research Portfolio, number 13507

WHO Dataset

Title of trial: Open Urethroplasty versus Endoscopic Urethrotomy (OPEN Trial) - Clarifying the management of men with recurrent urethral stricture: A pragmatic multicentre randomised superiority trial of open urethroplasty versus endoscopic urethrotomy

Trial Identifier: ISRCTN98009168

Date of ISRCTN assignation: 29/11/2012

Link to record in ISRCTN Register: <http://www.controlled-trials.com/ISRCTN98009168>

Protocol Version

Protocol version: 1.7

Protocol date: 17th February 2015

Funding

Funded by the UK Government National Institute for Health Research Health Technology Assessment Programme: project reference 10-57-23

Roles and Responsibilities

*Protocol contributions*

Rachel Stephenson: Trial Manager (January 2014 – March 2015); Newcastle Clinical Trials Unit, Newcastle University, Newcastle upon Tyne, UK

Sonya Carnell: Trial manager (March 2015 to date); Newcastle Clinical Trials Unit, Newcastle University, Newcastle upon Tyne, UK

Nicola Johnson: Assistant Trial Manager; Newcastle Clinical Trials Unit, Newcastle University, Newcastle upon Tyne, UK

Robbie Brown: Trial manager (May 2015 to date); Newcastle Clinical Trials Unit, Newcastle University, Newcastle upon Tyne, UK

Jennifer Wilkinson: Senior Trial Manager; Newcastle Clinical Trials Unit, Newcastle University, Newcastle upon Tyne, UK

Anthony Mundy: Trial conception and design, clinical support to sites; University College London Hospital, London, UK

Steven Payne: Trial conception and design; Central Manchester Foundation Trust, Manchester, UK

Nick Watkin: Trial conception and design, clinical support to sites and trial management group; St George’s Hospital, London, UK

James N’Dow: Trial conception and design, clinical support to sites; Academic Urology Unit, University of Aberdeen, UK

Andrew Sinclair: Trial planning including projected incidence estimates; Stepping Hill Hospital, Stockport, UK

Rowland Rees: Trial planning including site identification; Southampton General Hospital, Southampton, UK

Tim Rapley: Design and supervision of qualitative sub-study around feasibility

Stewart Barclay: Lay member of trial management group, review and revision of patient-facing documentation; Lay member of trial management group

Jonathan A Cook: Statistical advisor during trial design; Nuffield Department of Orthopaedics, Rheumatology and Musculoskeletal Sciences, University of Oxford, Oxford, UK

Beatriz Goulao: Junior statistician on project; Centre for Healthcare and Randomised Trials, University of Aberdeen, UK

Graeme MacLennan: Senior statistician on project; Centre for Healthcare and Randomised Trials, University of Aberdeen, UK

Gladys MacPherson: Design and management of trial database; Centre for Healthcare and Randomised Trials, University of Aberdeen, UK

Jing Shen: Design and conduct of health economic study and analyses; Institute of Health & Society, Newcastle University, Newcastle upon Tyne, UK

Luke Vale: Design and supervision of health economic study and analyses; Institute of Health & Society, Newcastle University, Newcastle upon Tyne, UK

Matthew Jackson: Assistance in writing trial protocol and trial publicity materials, design and validation of primary outcome measure; Institute of Cellular Medicine, Newcastle University, Newcastle upon Tyne, UK

John Norrie; Trial design, senior supervision of database management, statistical design and analysis; Centre for Healthcare and Randomised Trials, University of Aberdeen, UK

Elaine McColl: Trial design, protocol writing and senior supervision of trial management; Newcastle Clinical Trials Unit, Newcastle University, Newcastle upon Tyne, UK

Robert Pickard: Chief Investigator; Institute of Cellular Medicine, Newcastle University, Newcastle upon Tyne, UK

*Trial sponsor*

The Newcastle upon Tyne Hospitals NHS Foundation Trust, reference 6332.

Ms Amanda Tortice, Head, Research and Development, Joint Research Office

Regent Point, Regent Farm Road, Gosforth, Newcastle upon Tyne, NE3 3HD, UK

Email [Trust.R&D@nuth.nhs.uk](mailto:Trust.R&D@nuth.nhs.uk), Telephone +44 (0)1912825959, Fax +44 (0)1912824524

*Role of sponsor and funder*

The funder (through their peer and Funding Board review process) and the Sponsor through UK national approval mechanisms approved the study protocol but had no role in the collection, analysis, or interpretation of data, or writing of the report.

*Co-ordinating centre*

The trial was run by the Clinical Trials Unit based in Newcastle University, Newcastle upon Tyne, UK according to a written trial management plan approved by the funder. Data collection and management for the trial was carried out by the Centre for Healthcare and Randomised Trials based at the University of Aberdeen. Day-to-day responsibility for conducting the trial lay with the Trial Management Group which met monthly. The group consisted of the Chief Investigator, Trial Management Team, Data Management Team and additional involved people such as statistician and health economists as required.

*Trial steering committee*

The OPEN trial steering committee (TSC) consists of five independent members: Roger Kockelburg (independent clinician, chair), John Matthews (independent statistician), Alan McNeil (independent clinician), Howard Kynaston (independent clinician), Neil Campling (independent lay representative). The non-independent members include John Norrie, Gladys MacPherson, Graeme MacLennan, Elaine McColl, Robert Pickard, Jennifer Wilkinson, Rachel Stephenson, Sonya Carnell, and Nicola Johnson. The TSC meets at least every 6 months and has overall responsibility for supervision of conduct of the trial against the protocol and associated documents, and in line with Good Clinical Practice on behalf of the Sponsor and Funder.

*Data monitoring committee*

The OPEN data monitoring committee (DMC) comprises: Gordon Murray (statistician, chair), Richard Martin (methodologist), Thomas Pinkney (clinician). The focus of the DMEC is on safety and ethical issues. The DMC alone has access to unblinded comparative data and its role is to monitor these data and make recommendations to the TSC on whether there are any ethical or safety reasons why the trial should not continue. The DMC meets at least annually throughout the trial. The DAMOCLES charter is used as the basis for the DMC’s terms of reference.
